# Who benefits most from mild therapeutic hypothermia in coronary intervention era? A retrospective and propensity-matched study

**DOI:** 10.1186/cc9225

**Published:** 2010-08-16

**Authors:** Eisuke Kagawa, Ichiro Inoue, Takuji Kawagoe, Masaharu Ishihara, Yuji Shimatani, Satoshi Kurisu, Yasuharu Nakama, Kazuoki Dai, Takayuki Otani, Hiroki Ikenaga, Yoshimasa Morimoto, Kentaro Ejiri, Nozomu Oda

**Affiliations:** 1 Department of Cardiology, Hiroshima City Hospital, 7-33 Moto-machi, Naka-ku, Hiroshima 730-8518, Japan

## Abstract

**Introduction:**

The aim of the present study was to investigate the impact of the time interval from collapse to return of spontaneous circulation (CPA-ROSC) in cardiac arrest patients and the types of patients who will benefit from therapeutic hypothermia.

**Methods:**

Four hundred witnessed adult comatose survivors of out-of-hospital cardiac arrest of cardiac etiology were enrolled in the study. The favorable neurological outcome was defined as category 1 or 2 on the five-point Pittsburgh cerebral performance scale at the time of hospital discharge. A matching process based on the propensity score was performed to equalize potential prognostic factors in the hypothermia and normothermia groups, and to formulate a balanced 1:1 matched cohort study.

**Results:**

The rate of favorable neurological outcome was higher (*P *< 0.05) in the hypothermia group (*n *= 110) than in the normothermia group in patients with CPA-ROSC of 15 to 20 minutes (64% vs. 17%), 20 to 25 minutes (70% vs. 8%), 25 to 30 minutes (50% vs. 7%), 35 to 40 minutes (27% vs. 0%) and 40 to 45 minutes (29% vs. 2%). A similar association was observed in a propensity-matched cohort, but the differences were not significant. There was no significant difference in the rate of favorable neurological outcome between the hypothermia-matched group and the normothermia-matched group. In the patients whose CPA-ROSC was greater than 15 minutes, however, the rate of favorable neurological outcome was higher in the hypothermia-matched group than in the normothermia-matched group (27% vs. 4%, *P *< 0.001). In multivariate analysis, the CPA-ROSC was an independent predictor of favorable neurological outcome (every 1 minute: odds ratio = 0.89, 95% confidence interval = 0.85 to 0.92, *P *< 0.001).

**Conclusions:**

The CPA-ROSC is an independent predictor of neurological outcome. Therapeutic hypothermia is more beneficial in comatose survivors of cardiac arrest with CPA-ROSC greater than 15 minutes.

## Introduction

Cardiac arrest has a poor prognosis and is a major cause of unexpected death in developed countries. Despite cardiopulmonary resuscitation (CPR), only a few patients fully resume their former lifestyle, mainly because of anoxic brain injury [1,2]. Mild therapeutic hypothermia (MTH) improves neurological outcome in comatose survivors of cardiac arrest [3,4]. Previous studies have reported that coronary reperfusion therapy with percutaneous coronary intervention improves outcomes in out-of-hospital cardiac arrest (OHCA) patients [5-8]. The frequency of MTH is increasing in clinical settings; however, little is known about the types of patients who will benefit neurologically from MTH, will be able to resume their former lifestyle, and should not be treated with MTH [9].

The time interval from collapse to return of spontaneous circulation (ROSC) has been reported to be a strong independent predictor of neurological outcome in comatose survivors of cardiac arrest [4,10-14]. We therefore investigated the impact of the time interval from collapse to ROSC (CPA-ROSC) in OHCA patients treated with and without MTH.

## Materials and methods

### Study patients

We retrospectively enrolled witnessed adult (>18 years of age) OHCA patients with cardiac causes transported to Hiroshima City Hospital, who achieved ROSC and who were comatose between September 2003 and January 2010. All OHCA patients were treated in accordance with an advanced cardiac life support protocol, and the patients who met the criteria for hypothermia treatment were treated with MTH as reported previously [10]. MTH was fundamentally induced in cardiac arrest patients with presumed cardiac origin and the following criteria [15]: age 18 to 79 years, and an estimated interval of less than 15 minutes from collapse to the first attempt at resuscitation by any person.

Before 2006, an assessment of cardiac arrest complicated by ischemic heart disease was made in patients treated with and without MTH. In patients with suspected acute coronary syndrome, emergency coronary angiography or percutaneous coronary intervention, or both, were subsequently performed. After 2006, routine emergency coronary angiography was performed in patients treated with MTH.

The present study was approved by the local ethics committee on human research and is conducted in accordance with the guidelines of the Declaration of Helsinki. All data were collected within the normal daily care routine in an anonymous fashion. The institutional review board therefore waived the need for informed patient consent.

### Hypothermia protocol

MTH was induced in comatose survivors using a surface cooling mattress and the administration of physiological saline (4°C) as reported previously [10]. Before January 2008, the target temperature was set between 32°C and 34°C (fundamentally, 33°C) and was maintained for 48 hours followed by rewarming at 0.5°C every 12 hours. After January 2008, the core temperature was maintained for 24 hours and rewarming continued for 12 hours.

### Data collection

The primary endpoint was a favorable neurological outcome, which is defined as category 1 (good performance) or category 2 (moderate disability) on the five-point Pittsburgh cerebral performance scale; the other categories are 3 (severe disability), 4 (vegetative state), and 5 (death) at the time of hospital discharge [16].

### Statistical analysis

Continuous variables are presented as medians (with interquartile ranges), and categorical variables are presented as numbers and percentages. Differences between groups at baseline were analyzed using the Mann-Whitney U test for continuous variables and a chi-square test or Fisher's exact test for categorical variables as appropriate. The rate of favorable neurological outcome was plotted against the CPA-ROSC every 5 minutes for patients treated with and without MTH. We used Fisher's exact test to access differences in the rate of favorable neurological outcomes every 5 minutes between the groups.

To detect favorable neurological outcome, we constructed receiver-operating characteristic curves for the CPA-ROSC.

The threshold for performing MTH was set high early in the study period. For example, we induced MTH only in comatose survivors whose initial rhythm indicated ventricular fibrillation. Because the patients were not randomly assigned to receive MTH or normothermia, potential confounding and selection biases were accounted for by developing a propensity score. The propensity for MTH or not was determined without regard to the outcome using a multivariate regression model. For this model, we chose variables we thought might increase the propensity for MTH over normothermia therapy, which included age, Pittsburgh overall performance category score before cardiac arrest, initial rhythm, the time interval from collapse to start of CPR and the CPA-ROSC. This model yielded a concordance statistic of 0.80, which indicated good discrimination. A propensity score was then calculated from the logistic equation for each patient, which indicated the probability that a patient would be treated with MTH. All study patients were pooled and sorted according to their propensity scores in the ascending order and a propensity-matched cohort was formed [17].

A logistic regression model was used to examine the association between the CPA-ROSC and favorable neurological outcome in an unadjusted model, in a model adjusted for age, gender, and initial rhythm, and in a model adjusted for age, gender, Pittsburgh overall performance category score, initial rhythm, time interval from collapse to start of CPR, hypertension, diabetes mellitus, history of heart disease, emergency coronary angiography, primary percutaneous coronary intervention, use of intra-aortic balloon pump, use of extracorporeal life support, admission after 2006 and propensity score.

The JMP statistical package (version 5.0.1 J; SAS Institute, Cary, NC, USA) and R version 2.9.2 [18] were used for statistical analyses. All tests were two-sided, and P < 0.05 was considered statistically significant.

## Results

### Patient characteristics

**Figure 1:**
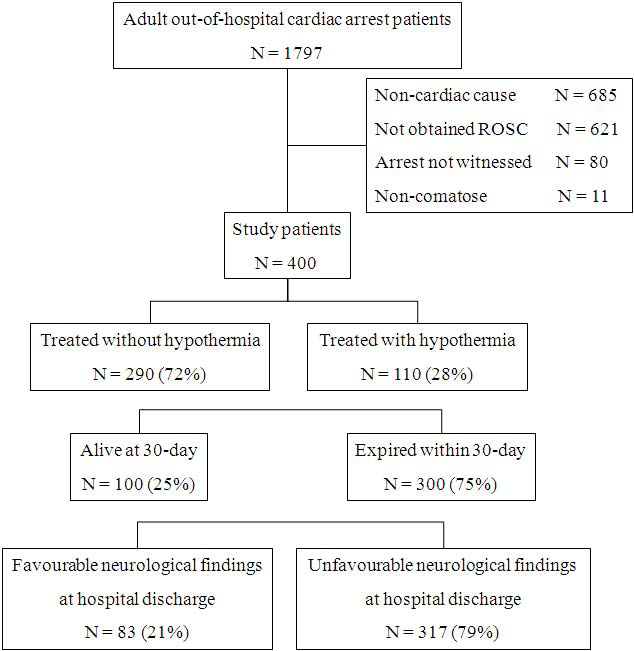
**Flow diagram of study patients and outcomes**. ROSC, return of spontaneous circulation.

A flow diagram of the study patients and their outcomes is depicted in Figure 1. Witnessed adult comatose survivors of OHCA (*n *= 400) with cardiac causes were enrolled in the study. The baseline clinical characteristics, treatment and findings for study patients are shown in Tables 1 and 2. One hundred and ten patients (28%) were treated with MTH (hypothermia group) and 290 patients were treated without MTH (normothermia group). There were many differences in baseline characteristics between these groups. The normothermia group was in a more severe condition for resuscitation.

**Table 1:** Baseline clinical characteristics of the study patients

	Hypothermia group (*n *= 110)	Normothermia group (*n *= 290)	*P *value
Age (years)	55 (46 to 68)	74 (62 to 83)	<0.001
Male gender	90 (82)	177 (61)	<0.001
Pittsburgh overall performance category scale before index cardiac arrest			<0.001
Category 1	109 (99)	216 (74)	
Category 2	1 (1)	62 (21)	
Category 3	0 (0)	12 (4)	
Category 4	0 (0)	0 (0)	
Initial rhythm			<0.001
Ventricular fibrillation	64 (58)	41 (14)	
Pulseless electrical activity	23 (21)	134 (46)	
Asystole	23 (21)	115 (40)	
Bystander cardiopulmonary resuscitation	61 (55)	137 (47)	0.14
Time interval from collapse to start of cardiopulmonary resuscitation (minutes)	6 (1 to 10)	7 (1 to 14)	0.049
Time interval from collapse to return of spontaneous circulation (minutes)	35 (21 to 48)	43 (23 to 56)	<0.01
Admission after 2006	83 (75)	169 (58)	<0.01
Hypertension	44 (40)	85 (29)	0.04
Diabetes mellitus	29 (26)	47 (16)	0.02
Previous history of heart disease	42 (38)	63 (22)	<0.001
Previous history of myocardial infarction	18 (16)	15 (5)	<0.001
Previous history of chronic heart failure	16 (15)	27 (9)	0.13
Cause of cardiac arrest			
Acute coronary syndrome	46 (41)	56 (19)	
Old myocardial infarction	12 (11)	4 (1)	
Cardiomyopathy	13 (12)	8 (3)	
Spasm of coronary artery	8 (7)	2 (1)	
Others	27 (25)	72 (25)	
Unknown	4 (4)	148 (51)	

Data presented as median (interquartile range) or *n *(%).

**Table 2:** In-hospital treatment and findings in the study patients

	Hypothermia group (*n *= 110)	Normothermia group (*n *= 290)	*P *value
Dose of epinephrine (mg)	1 (0 to 3)	1 (0 to 2)	0.54
Emergency coronary angiography	68 (62)	34 (12)	<0.001
Primary percutaneous coronary intervention	33 (30)	16 (6)	<0.001
Use of intra-aortic balloon pump	65 (59)	22 (8)	<0.001
Use of extracorporeal life-support	27 (25)	13 (4)	<0.001
Time interval from return of spontaneous circulation to target temperature (minutes)	149 (104 to 262)		
Protocol of duration of cooling/rewarming 24 hours/12 hours	40 (36)		
Duration of cooling (hours)	47 (24 to 49)		
Duration of rewarming (hours)	47 (17 to 57)		
Duration of hospital stay (days)	17 (5 to 29)	1 (1 to 3)	<0.001
Cause of death within 30 days			
Hemodynamic instability	21 (36)	90 (37)	
Withdrawal of intensive therapy	18 (32)	85 (35)	
Multiple organ failure	15 (26)	10 (4)	
Pneumonia	3 (6)	12 (5)	
Other	0 (0)	46 (19)	

Data presented as median (interquartile range) or *n *(%).

### Outcomes

The outcomes of the study patients are shown in Table 3. The rate of favorable neurological outcome (39% vs. 14%, *P *< 0.001) and 30-day survival (48% vs. 16%, *P *< 0.001) were higher in the hypothermia group than in the normothermia group. The CPA-ROSC in patients with a favorable neurological outcome was longer (*P *< 0.001) in the hypothermia group (median, 22 minutes; interquartile range, 16 to 32 minutes; mean ± standard deviation, 25 ± 13 minutes) than in the normothermia group (median, 11 minutes; interquartile range, 5 to 14 minutes; mean ± standard deviation, 12 ± 8 minutes).

**Table 3:** Outcomes

	Hypothermia group (*n *= 110)	Normothermia group (*n *= 290)
Favorable neurological outcome	43 (39)	40 (14)
Good cerebral performance	42 (38)	39 (13)
Moderate cerebral disability	1 (1)	1 (1)
Severe cerebral disability	0 (0)	1 (1)
Coma or vegetative state	8 (7)	8 (3)
Dead	59 (54)	241 (82)
Thirty-day survival	53 (48)	47 (16)

Data presented as *n *(%).

**Figure 2:**
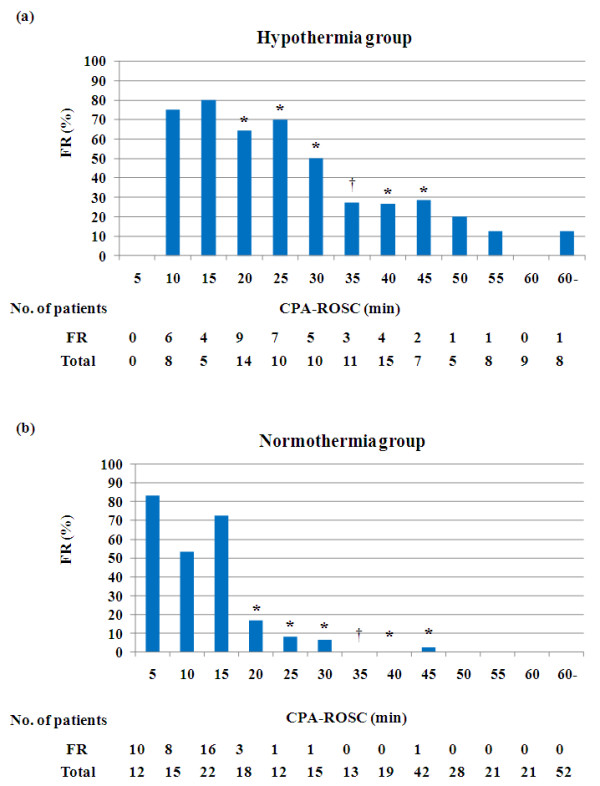
**Rate of favorable neurological outcome**. The rate of favorable neurological outcome (FR) by the time interval from collapse to return of spontaneous circulation (CPA-ROSC) every 5 minutes in **(a) **the hypothermia group and **(b) **the normothermia group. *The rate of FR in the hypothermia group was higher than that in the normothermia group (*P *< 0.05). ^†^There was a trend toward a higher rate of FR in the hypothermia group than in the normothermia group (0.05 <*P *< 0.10).

The rate of favorable neurological outcome was plotted against the CPA-ROSC every 5 minutes (Figure 2). In the hypothermia group, the rate of favorable neurological outcome decreased in a stepwise fashion when the CPA-ROSC was longer than 25 minutes. In the normothermia group, the rate of favorable neurological outcome decreased remarkably when the CPA-ROSC was longer than 15 minutes, and none of the patients whose CPA-ROSC was longer than 45 minutes had a favorable neurological outcome. The rate of favorable neurological outcome was significantly higher in the hypothermia group than in the normothermia group in patients with a CPA-ROSC of 15 to 20 minutes (64% vs. 17%, *P *< 0.01), 20 to 25 minutes (70% vs. 8%, *P *< 0.01), 25 to 30 minutes (50% vs. 7%, *P *= 0.02), 35 to 40 minutes (27% vs. 0%, *P *= 0.03) and 40 to 45 minutes (29% vs. 2%, *P *= 0.049). There was a trend toward a higher rate of favorable neurological outcome in the hypothermia group than in the normothermia group in patients in whom the CPA-ROSC was 30 to 35 minutes (27% vs. 0%, *P *= 0.08). The rate of favorable neurological outcome was similar in patients with a CPA-ROSC less than 15 minutes.

**Figure 3:**
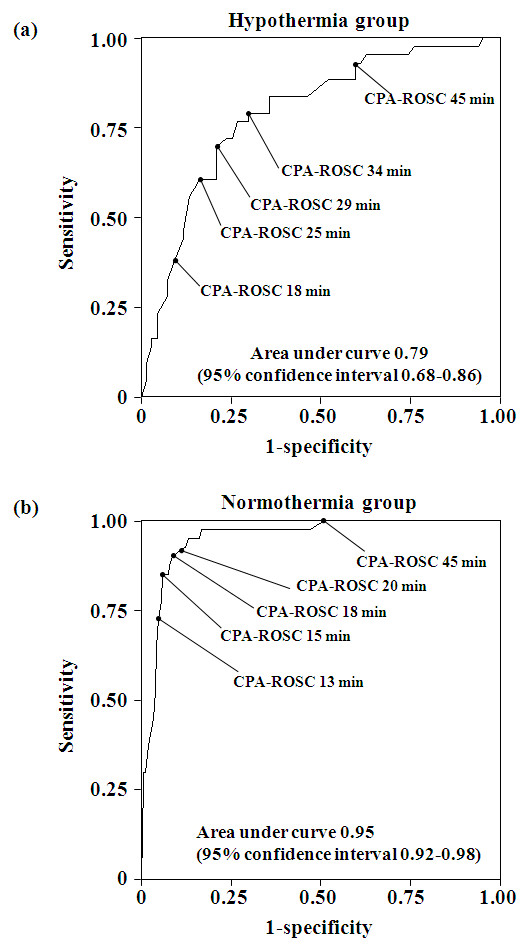
**Time interval from collapse to return of spontaneous circulation predicting favorable neurological outcome**. Receiver-operating characteristic curves for predicting a favorable neurological outcome in **(a) **the hypothermia group and **(b) **the normothermia group. CPA-ROSC, time interval from collapse to return of spontaneous circulation.

Receiver-operating characteristic curves of the CPA-ROSC to detect favorable neurological outcome are shown in Figure 3. The areas under the receiver-operating characteristic curve were 0.79 (95% confidence interval (CI) = 0.68 to 0.86) and 0.95 (95% CI = 0.92 to 0.98) in the hypothermia and normothermia groups, respectively. The cut-off values for the CPA-ROSC of 29 minutes in hypothermia group and 15 minutes in the normothermia group had the highest combined sensitivity and specificity, with accuracy of 75.4% and 92.8% for identifying favorable neurological outcomes, respectively (Table 4). A long CPA-ROSC was associated with more accurate negative predictive values.

**Table 4:** Cut-off values for CPA-ROSC and diagnostic accuracy

CPA-ROSC	Sensitivity	Specificity	Positive predictive value	Negative predictive value	Accuracy (%)
Hypothermia group					
18 minutes	37 (28 to 46)	91 (86 to 96)	73 (65 to 81)	69 (60 to 78)	70
25 minutes	60 (51 to 69)	84 (77 to 91)	70 (61 to 79)	77 (69 to 85)	74
29 minutes	70 (61 to 79)	79 (71 to 87)	68 (59 to 77)	80 (73 to 87)	75
34 minutes	79 (71 to 87)	70 (61 to 79)	63 (54 to 72)	84 (77 to 91)	74
45 minutes	93 (88 to 98)	40 (31 to 49)	50 (41 to 59)	90 (84 to 96)	61
65 minutes	100	4 (1 to 8)	40 (31 to 49)	100	42
Normothermia group					
13 minutes	73 (68 to 78)	95 (92 to 98)	71 (66 to 76)	96 (94 to 98)	92
15 minutes	85 (81 to 89)	94 (91 to 97)	69 (64 to 74)	98 (96 to 99)	93
18 minutes	90 (87 to 93)	91 (88 to 94)	62 (56 to 68)	98 (96 to 99)	91
20 minutes	92 (89 to 95)	88 (84 to 92)	55 (49 to 61)	99 (98 to 99)	89
45 minutes	100	49 (43 to 55)	24 (19 to 29)	100	56

Data presented as mean (95% confidence interval) (%). Time interval from collapse to return of spontaneous circulation (CPA-ROSC) cut-off value of 29 minutes in the hypothermia group and 15 minutes in the normothermia group had the highest combined sensitivity and specificity, with accuracies of 75.4% and 92.8%, respectively.

### Propensity matched analysis

**Figure 4:**
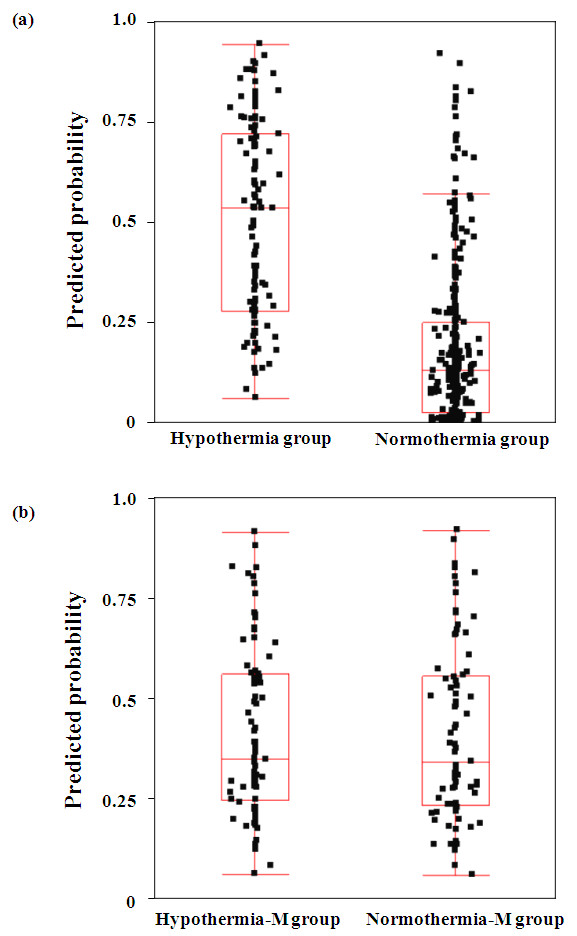
**Distribution of propensity scores**. Distribution of propensity scores **(a) **in the hypothermia and normothermia groups and **(b) **in the matched hypothermia-M and normothermia-M groups.

The propensity score-matching process selected 79 patients from the hypothermia group (hypothermia-M group) and 79 patients from the normothermia group (normothermia-M group). The mean ± standard deviation propensity score was 0.41 ± 0.22 in the hypothermia-M group and was 0.41 ± 0.22 in the normothermia-M group (*P *= 0.94; Figure 4). Propensity-matched patient characteristics, treatments and outcomes are presented in Table 5. Baseline characteristics were similar in the two groups except for emergency coronary angiography, primary percutaneous coronary intervention, use of intra-aortic balloon pump and use of extracorporeal circulation. In patients with a favorable neurological outcome, the CPA-ROSC was significantly longer (*P *< 0.001) in patients in the hypothermia-M group (median, 22 minutes; interquartile range, 16 to 31 minutes; mean ± standard deviation, 25 ± 12 minutes) than those in the normothermia-M group (median, 10 minutes; interquartile range, 5 to 13 minutes; mean ± standard deviation, 11 ± 9 minutes).

**Table 5:** Patient characteristics and outcomes of propensity-matched patients

	Hypothermia-M group (*n *= 79)	Normothermia-M group (*n *= 79)	*P *value
Age (years)	60 (49 to 72)	61 (50 to 71)	0.62
Male gender	62 (78)	55 (70)	0.20
Pittsburgh overall performance category scale 1 before index cardiac arrest	78 (99)	79 (100)	>0.99
Initial rhythm			0.72
Ventricular fibrillation	23 (29)	25 (32)	
Pulseless electrical activity	35 (44)	30 (38)	
Asystole	21 (27)	24 (30)	
Bystander cardiopulmonary resuscitation	42 (53)	37 (47)	0.43
Time interval from collapse to start of cardiopulmonary resuscitation (minutes)	8 (1 to 12)	5 (1 to 10)	0.29
Time interval from collapse to return of spontaneous circulation (minutes)	35 (22 to 49)	39 (14 to 50)	0.86
Hypertension	36 (46)	25 (32)	0.07
Diabetes mellitus	23 (29)	16 (20)	0.20
Previous history of heart disease	31 (39)	20 (25)	0.06
Previous history of myocardial infarction	12 (15)	5 (6)	0.07
Previous history of chronic heart failure	12 (15)	9 (11)	0.48
Cause of cardiac arrest			
Acute coronary syndrome	35 (44)	20 (25)	
Old myocardial infarction	8 (10)	7 (9)	
Cardiomyopathy	8 (10)	4 (5)	
Spasm of coronary artery	4 (5)	1 (1)	
Others	18 (23)	14 (21)	
Unknown	6 (8)	33 (42)	
Emergency coronary angiography	44 (56)	20 (25)	<0.01
Primary percutaneous coronary intervention	23 (29)	9 (11)	<0.01
Use of intra-aortic balloon pump	44 (56)	15 (19)	<0.001
Use of extracorporeal life-support	21 (27)	5 (6)	<0.001
Duration of hospital stay (days)	16 (4 to 27)	1 (0 to 11)	<0.001
Favorable neurological outcome	24 (30)	23 (29)	0.86
Good cerebral performance	24 (30)	23 (29)	
Coma or vegetative state	6 (8)	1 (1)	
Dead	49 (62)	55 (70)	
Thirty-day survival	32 (41)	23 (29)	0.13

Data presented as median (interquartile range) or *n *(%).

**Figure 5:**
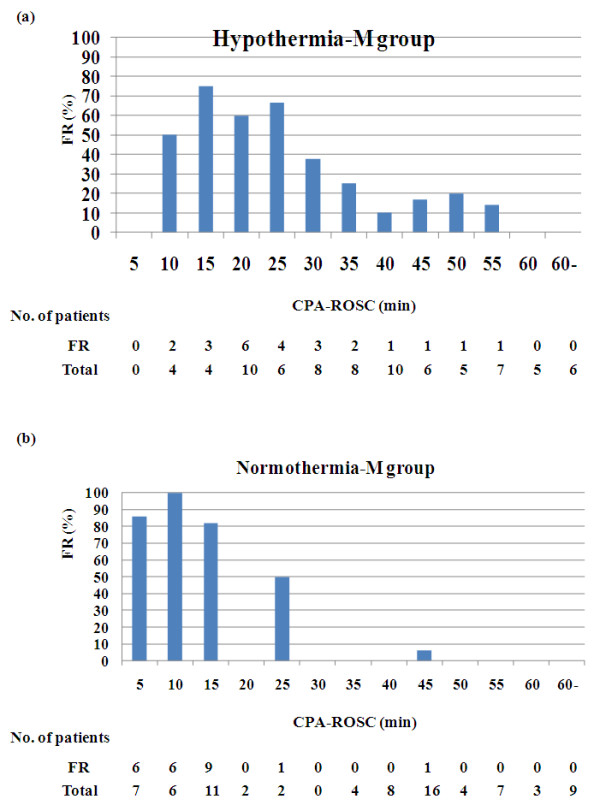
**Rate of favorable neurological outcome in the propensity-matched cohort**. Plot representing the rate of favorable neurological outcome (FR) against the time interval from collapse to return of spontaneous circulation (CPA-ROSC) every 5 minutes in **(a) **the propensity-matched hypothermia-M group and **(b) **the normothermia-M group.

The rate of favorable neurological outcome was plotted against the CPA-ROSC every 5 minutes in the propensity-matched cohort (Figure 5). An association similar to the full cohort (Figure 2) was observed in the propensity-matched cohort, but the difference was not statistically significant and might be caused from the small sample size of the study. There was no significant difference in the rate of favorable neurological outcome between the hypothermia-M group and the normothermia-M group in the entire propensity-matched cohort (30% vs. 29%, *P *= 0.86). In patients whose CPA-ROSC was more than 15 minutes, however, the rate of favorable neurological outcome was higher in the hypothermia-M group than in the normothermia-M group (27% vs. 4%, *P *< 0.001).

### Predictor of neurological outcome

In the full cohort, we tested the association between the CPA-ROSC and favorable neurological outcome in multiple models. In an unadjusted model, there was a significant association between the CPA-ROSC and favorable neurological outcome (every 1 minute: odds ratio = 0.90, 95% CI = 0.88 to 0.92, *P *< 0.001). After adjustment for age, gender, and initial rhythm, this association was still significant (odds ratio = 0.89, 95% CI = 0.86 to 0.92, *P *< 0.001). Finally, after adjustment for all of the above-mentioned variables, the association between the CPA-ROSC and favorable neurological outcome remained significant (odds ratio = 0.89, 95% CI = 0.85 to 0.92, *P *< 0.001).

## Discussion

We showed that the CPA-ROSC is a strong independent predictor of favorable neurological outcome in the present MTH era, and that MTH prolongs the maximum CPA-ROSC to obtain a favorable neurological outcome. MTH is more beneficial in OHCA patients with a CPA-ROSC longer than 15 minutes in terms of neurological outcome.

As there is not enough blood flow to maintain the metabolism of organs in cardiac arrest patients, despite chest compression, the organs experience ischemic damage from the time of collapse to ROSC [19]. Furthermore, cardiac arrest triggers neuronal death and inflammation, as well as mitochondrial dysfunction, oxidative stress, altered signal transduction and programmed cell death, which are implicated in delayed injury after reperfusion. It has been suggested that the longer the CPA-ROSC, the greater the damage to organs, including the brain [20]. We showed that MTH may protect organs from these delayed injuries. We also showed that the rate of favorable neurological outcome in OHCA patients with a CPA-ROSC greater than 15 minutes was terrible, but was extended to 30 minutes or more in patients treated with hypothermia. MTH may protect organs from delayed injury, but the no perfusion or low perfusion state is longer, and damage of organs may be too severe for patients to return to their former lifestyle. The metabolic phase of the three-phase model explains these 15 minutes well [20].

The International Liaison Committee on Resuscitation has issued guidelines for treatment with MTH [21]. Because of the human resources and costs associated with MTH, the criteria for treatment are different in each hospital [3,4,8,10]. The type of patients who can resume their former lifestyle without MTH has not been discussed. The results of the present study suggest that MTH may be more beneficial in patients with a CPA-ROSC greater than 15 minutes and a negative predictive value of 100% than in those with a CPA-ROSC of 45 minutes in OHCA patients without MTH. We suggest that comatose survivors of cardiac arrest of cardiac origin with a CPA-ROSC greater than 15 minutes must be treated with MTH. On the contrary, the neurological outcome of patients with a CPA-ROSC less than 15 minutes is likely to be similar with or without MTH. This does not, however, mean that OCHA patients with a CPA-ROSC less than 15 minutes should not be treated with MTH. Our study investigated only the neurological outcomes at the time of hospital discharge, and so performing MTH to protect mild neurological or organ damage should be permitted.

In our study, the CPA-ROSC of 65 minutes in the hypothermia group and of 45 minutes in the normothermia group had a negative predictive value of 100%. These values may be the present points of no return for CPR in the present clinical settings. Our study showed that the shorter the CPA-ROSC, the higher the ratio of favorable neurological outcome. Cardiac arrest patients should be treated in a manner that achieves ROSC as soon as possible to obtain a better outcome.

### Study limitations

Our study was not double-blind or randomized and had the inherent limitations of any single-centre retrospective investigation. The present study was prone to biases related to unmeasured factors. We did, however, use multivariate and propensity analyses to carefully match patients in an effort to eliminate bias. It was difficult for us to estimate the time of collapse of cardiac arrest patients correctly, so we enrolled only the witnessed cardiac arrest patients in this study. CPR guidelines were changed during the study period, which might affect the outcomes. We attempted to adjust for this effect with the additional covariate of admission after 2006 [12]. Although we found statistically significant differences between the groups in our main cohort, we found no statistically significant differences between groups in our propensity-matched cohort. This difference might be caused by a sample that was small and may have been subject to type II error, and by good results for patients whose CPA-ROSC was less than 5 minutes in the normothermia-M group with no one whose CPA-ROSC was less than 5 minutes treated with hypothermia. We thought that clinical signs, such as neurological findings, gasping and spontaneous respiration, might be associated with the propensity score [22]. Although these clinical signs should have been recorded at fixed times after ROSC for proper analysis, we were unable to retrieve such data from the patients' medical records.

## Conclusions

The CPA-ROSC is an independent predictor of neurological outcome in comatose survivors of OHCA. A CPA-ROSC longer than 15 minutes in the normothermia therapy group and longer than 30 minutes in the hypothermia therapy group was associated with low rates of favorable neurological outcome. MTH prolongs the CPA-ROSC, which may help comatose survivors of cardiac arrest obtain favorable neurological outcomes, and is more beneficial in patients whose CPA-ROSC is greater than 15 minutes.

## Key messages

• MTH is more beneficial in patients whose CPA-ROSC is greater than 15 minutes.

• Neurological outcome in patients treated with normothermia and hypothermia were similar in patients with a CPA-ROSC less than 15 minutes.

• The CPA-ROSC is an independent predictor of neurological outcome in comatose survivors of cardiac arrest.

• A CPA-ROSC greater than 15 minutes in the normothermia therapy group and of 30 minutes in the hypothermia therapy group was associated with low rates of favorable neurological outcome.

## Abbreviations

CI: confidence interval; CPA-ROSC: time interval from collapse to return of spontaneous circulation; CPR: cardiopulmonary resuscitation; MTH: mild therapeutic hypothermia; OHCA: out-of-hospital cardiac arrest; ROSC: return of spontaneous circulation.

## Competing interests

The authors declare that they have no competing interests.

## Authors' contributions

All authors participated in the design and coordination of the study and draft of the manuscript. All authors read and approved the final manuscript.
